# Knowledge of Alzheimer's Disease Among Medical Students of a Medical College

**Published:** 2018-06-30

**Authors:** Rachana Sharma, Subhash Chandra Sharma, Sudarshan N Pradhan, Pratikshya Chalise, Luna Paudel

**Affiliations:** 1 Department of Psychiatry, Kathmandu Medical College, Sinamangal, Kathmandu, Nepal

**Keywords:** *alzheimer's disease*, *dementia*, *knowledge*, *medical students*

## Abstract

**Introduction:**

Dementia is a public health concern as the prevalence is increasing worldwide with significant increase being in low-middle income countries. However these countries appear to be less prepared in handling this rise in terms of diagnosis and management.

**Methods:**

This cross-sectional study was conducted in Kathmandu medical College, from June 2017 to July 2017. Purposive sampling was done and the medical students who were in their first and final year of study were included in the study after obtaining an informed consent.

**Results:**

Total 185 students were included in the study, 80 (43.24%) from first year and 105 (56.75%) from final year of medical study. The mean score of knowledge among the students was 17.44+2.46; 15.32+1.22 among first year and 19.06+1.87 among the final year students. Majority of the students said they have heard about dementia however, only 3 (3.75%) of first year and 43 (41.9%) from final year students have either attended a class or continuing medical education on dementia

**Conclusions:**

The knowledge about dementia was found to be average among medical students but better among final year medical students in comparison to first year. The knowledge was found to be better statistically among those who had either attended a class or continuing medical education on dementia or had exposure through different medias.

## INTRODUCTION

Dementia is a growing public health problem and the prevalence of dementia is rising globally with significant increase being in low-middle income countries.^1^ Epidemiological survey on dementia hasn't been done in Nepal. However, two thesis done by psychiatry resident in a tertiary level hospital of eastern Nepal, found the prevalence of dementia to be around 12% in population above 60 years.^2–3^ However the health care system of these countries are less prepared to handle these rise in numbers of patients with dementia in terms of diagnosis and management.^4^

In context of Nepal, there is lack of specialized care specialist thus major responsibility for diagnosis and management lies on the medical graduates. However medical schools in Nepal provide minimal training in geriatric medicine and dementia care in undergraduate level,^5^ and thus the knowledge of medical students regarding dementia can be presumed to be limited.

The study was designed to assess the knowledge of first and final year medical students regarding Alzheimer's Disease.

## METHODS

This cross-sectional study was carried out among the first and final year medical students of Kathmandu Medical College from June 2017 to July 2017. Approval for the study was obtained from Ethical committee and Institutional Review Committee of Kathmandu Medical College. The students were approached in person in their class and explained briefly about the study. The consenting students meeting our research criteria were enrolled in the study. The inclusion criteria of the study were: i) Those students who are either in first year or final year of medical school ii) Those students who agree to participate in the study.

Self-designed, semi-structured proforma was used to collect the background information like socio-demographic profile of the students and some information regarding dementia. Alzheimer's Disease knowledge Scale (ADKS) was used to assess the students' knowledge on different aspects of Dementia. ADKS was used because of its ease to use, demonstrated reliability and validity and applicability for different groups of participants (health professionals, general public and caregivers). The ADKS consists of 30 true/ false items with the resulting score being the numbers answered correctly. Reliability of the ADKS measured by test-retest correlation is 0.81; internal consistency, as measured by the average inter-item correlation of [ is 0.71 and content/ predictive validity is adequate. The data was analyzed using SPSS version 20.^6^

## RESULTS

In this study, total of 185 students both from first and final year participated. The students were from 18 years to 26 years of age and the mean age was 21.16±4.86 years. Seventeen individuals (9.2%) said they had some family members or friends suffering from dementia, among them, 6.5% said their close relatives were suffering from dementia (Grandmother and Grandfather), 0.5% said their distant relative had dementia and the remaining didn't specify. Other relevant socio-demographic details of the participants are given (Table 1).

**Table 1.** Sociodemographic profile of participants.

Variable		n (%)
Gender	Male	115 (62.2)
	Female	70 (37.8)
Year of Medical School	First	80 (43.2)
	Final	105 (56.8)
Ever heard about Dementia	Yes	176 (95.1)
	No	9 (4.9)
Ever attended classes or CME on Dementia	Yes	47 (25.4)
	No	138 (74.6)
Ever been educated about Dementia	Yes	86 (46.5)
	No	99 (53.5)
Family or friends suffering from Dementia	Yes	17 (9.2)
	No	168 (90.8)
Ever studied about Dementia on your own	Yes	70 (37.8)
	No	115 (62.2)

Students were asked to self-rate their knowledge on dementia on a scale of 0-10. Among 185 students, 174 (94.05%) rated their knowledge whereas 11 students (5.95%) didn't mention anything. The total mean score among 174 students was 3.60±1.87, minimum being 0 and maximum being 10. The mean score among first year students was 2.99±1.74, whereas it was 4.04±1.85 among final year students. The difference between self-rated knowledge was found to be statistically significant. Students knowledge was further assessed using Alzheimer's Disease Knowledge Scale (ADKS). The mean score among 185 students was 17.44±2.46; the mean score among first year students was 15.32±1.22 whereas that among final year students was 19.06±1.87. The difference between first and final year score was found to be statistically significant. The difference in knowledge among first and final year students (both self-rated and according to ADKS) was also found to be statistically significant according to Spearman's correlation. Other relevant difference among first and final year students are given (Table 2).

**Table 2.** Year of Study vs Different Variables.

		First Year n (%)	Final Year n (%)	P
Gender	Male	51 (27.6%)	64 (34.6%)	
	Female	29 (15.7%)	41 (22.2%)	0.408
Ever heard	Yes	74 (40%)	102 (55.1%)	
about dementia	No	6 (3.2%)	3 (1.6%)	0.134
Educated about dementia	Yes	23 (12.4%)	63 (34.1%)	
	No	57 (30.8%)	42 (22.7%)	0.000
Ever studied about dementia on your own	Yes	33 (17.8%)	37 (20%)	
	No	47 (25.4%)	68 (36.8%)	0.247
			
Ever attended any classes or CME on dementia	Yes	3 (1.6%)	44 (23.8%)	
	No	77 (41.6%)	61 (33.0%)	0.000

A mong the 80 first year and 105 final year medical students, 74 (92.5%) and 102 (97.14%) respectively said that they have heard about dementia through various sources. However, the difference was not found to be statistically significant. Similarly 3 (3.75%) of 80 first year students and 43 (41.9%) of 105 final year students said that they have either attended a class or CME on dementia and the difference was found to be statistically significant. Most of the students 115 (62.2% including both first and final year) said that they haven't studied about dementia on their own and the difference between first and final year students was not statistically significant. However, when asked whether they have been educated about dementia via any means, 63 (60%) final year students and 23 (28.75%) first year students responded 'Yes' and the difference was statistically significant. The two factors that were found to be significantly associated with better knowledge about dementia among medical students were attending class or CME and being educated about dementia via different media (P = 0.000).

## DISCUSSION

This study was conducted with the primary objective of evaluating the knowledge of medical students (first and final year) regarding dementia. In the study it was found that final year medical students had better knowledge both self-rated and according to ADKS than first year, meaning that those who self-rated their knowledge to be good scored well on ADKS as well. Similar finding was noted in a study conducted among medical students in US^7^ as well as in a study conducted in Queensland, Australia among different health care staffs.^8^

Out of 30 questions first year students on average answered 50% questions correctly whereas final years score was 63% on average. In an average, the correct response was just above 50% among first and final year students, which can be said to be limited if not poor. The knowledge was found to be better (statistically significant) among those who had either been educated about dementia via different means like newspaper, television, radio etc and among those who have attended either class or CME on dementia. In a study conducted among different health care staffs in Australia, found the average percentage of correct response among participants to be 79%^8^ which was higher than our study. One possible reason to this might be Australia being developed nation has better access to various media compared to ours. The other possible reason might be the sample population. Australian study included medical professionals who were directly involved in treating and caring the patient whereas our study only included medical students who had no or limited contact with patients with dementia.

In a similar study conducted among final and first year medical students of various medical schools of United States showed similar results like ours in which final year students scored relatively better than first year and those students whose family member or friends had AD were more likely to perform well.^7^ However, in our study having a family member or friend with dementia didn't affect the knowledge of the students. The possible reason for this might be because our students mightn't have been directly involved in taking care of the family member suffering from dementia.

Our study revealed positive correlation between knowledge about dementia and self- rated dementia knowledge and having attended dementia related class or media exposure. Our finding is in accordance to the study conducted in Australia among health care staff (clinical and non-clinical) which showed those who have attended dementia related class had better knowledge.^8^ In another similar study conducted in Hong Kong also revealed that attending more hours of class was related to the better level of knowledge of dementia.^9^ Similarly another study conducted in the United State among various groups also concluded that attending dementia related classes and exposure to multiple information sources enhanced the knowledge regarding dementia. ^10^ Both, attending more hours and getting exposed to various media regarding dementia is a positive attribute to improving knowledge.

One of the major limitations of this study was that it assessed the knowledge of medical students of a single medical college. We have various medical colleges under different universities in our country which have their own curriculum. A multi-centric study among students of various medical colleges would give a better picture of the scenario. Nevertheless, through this study finding I would like to emphasize on the need to improve the knowledge of medical students regarding dementia as in a developing country like ours WHO has estimated the burden of disease to be high and medical graduates are the primary contact for patients initially. So, preparation of medical graduates is of utmost importance.

## CONCLUSIONS

According to this study, final year medical students were found to have better knowledge regarding dementia than first year students though the overall knowledge was found to be just above average. The knowledge was found to be better among those who had attended either CME or any classes on dementia and among those who had media exposure regarding the same.
